# GWAS and Transcriptomic Analysis Identify *OsRING315* as a New Candidate Gene Controlling Amylose Content and Gel Consistency in Rice

**DOI:** 10.1186/s12284-024-00718-8

**Published:** 2024-06-08

**Authors:** Shuai Nie, Luo Chen, Minhua Zheng, Jingfang Dong, Yamei Ma, Lian Zhou, Jian Wang, Jiansong Chen, Haifei Hu, Tifeng Yang, Junliang Zhao, Shaohong Zhang, Wu Yang

**Affiliations:** Rice Research Institute, Guangdong Academy of Agricultural Sciences, Guangdong Key Laboratory of New Technology in Rice Breeding, Guangdong Rice Engineering Laboratory, Key Laboratory of Genetics and Breeding of High Quality Rice in Southern China (Co-construction by Ministry and Province), Ministry of Agriculture and Rural Affairs, Guangzhou, 510640 P.R. China

**Keywords:** Rice, Cooking quality, Genome-wide association study, Quantitative trait loci (QTL), Candidate gene

## Abstract

Cooking quality is the main factor determining the market value of rice. Although several major genes and a certain number of QTLs controlling cooking quality have been identified, the genetic complexity and environmental susceptibility limit the further improvement for cooking quality by molecular breeding. This research conducted a genome-wide association study to elucidate the QTLs related to cooking quality including amylose content (AC), gel consistency (GC) and alkali spreading value (ASV) by using 450 rice accessions consisting of 300 *indica* and 150 *japonica* accessions in two distinct environments. A total of 54 QTLs were identified, including 25 QTLs for AC, 12 QTLs for GC and 17 QTLs for ASV. Among them, 10 QTLs were consistently observed by the same population in both environments. Six QTLs were co-localized with the reported QTLs or cloned genes. The *Wx* gene for AC and GC, and the *ALK* gene for ASV were identified in every population across the two environments. The *qAC9-2* for AC and the *qGC9-2* for GC were defined to the same interval. The *OsRING315* gene, encoding an E3 ubiquitin ligase, was considered as the candidate gene for both *qAC9-2* and *qGC9-2*. The higher expression of *OsRING315* corresponded to the lower AC and higher GC. Three haplotypes of *OsRING315* were identified. The Hap 1 mainly existed in the *japonica* accessions and had lower AC. The Hap 2 and Hap 3 were predominantly present in the *indica* accessions, associated with higher AC. Meanwhile, the GC of accessions harboring Hap 1 was higher than that of accessions harboring Hap 3. In addition, the distribution of the three haplotypes in several rice-growing regions was unbalanced. The three traits of cooking quality are controlled by both major and minor genes and susceptible to environmental factors. The expression level of *OsRING315* is related to both AC and GC, and this gene can be a promising target in quality improvement by using the gene editing method. Moreover, the haplotypes of *OsRING315* differentiate between *indica* and *japonica*, and reveal the differences in GC and AC between *indica* and *japonica* rice.

**Supplementary Information:**

The online version contains supplementary material available at 10.1186/s12284-024-00718-8.

## Introduction

Rice is one of the world’s most important food crops, feeding nearly half of the world’s population. Improving rice yield and quality is the major goal of rice breeding. With the improvement of people’s living standards, high quality rice is the primary concern of rice breeders and consumers. Rice quality mainly includes milling quality, appearance quality and cooking quality (Li et al. 2022). Among them, cooking quality reflects the characteristic and palatability of cooked rice, which is directly related to the taste of rice. Amylose content (AC), gel consistency (GC) and gelatinization temperature (GT) are often used as the major indexes to evaluate cooking quality. AC is considered to be the major predictor of cooking quality (Ramesh et al. 1999). In general, AC is positively correlated with the hardness of cooked rice. According to AC, rice varieties are divided into five types: waxy (0–2%), very low (3–9%), low (10–19%), intermediate (20–25%) and high (> 25%) (Juliano 1992). However, some varieties with the same AC vary greatly in hardness of their cooked rice. This may be related to the difference between GC and GT. GC is a criterion for evaluating cooked rice texture and indicated by the distance of continuous movement of the rice flour gel after cooling (Cagampang et al. 1973). GC is generally divided into three levels: hard (< 40 mm), mediate (41–60 mm) and soft (> 61 mm) (Tang et al. 1991). GT is defined as the temperature at which almost all starch particles with a semi-crystalline structure begin to melt and lose birefringence in hot water. GT is usually assessed by alkali spreading value (ASV). ASV refers to the erosion degree of alkali to head milled rice grains. ASV can be divided into 1–7 grades, which are the opposite of GT, with grades 1 to 3 corresponding to high GT (> 74 °C), grades 4 and 5 corresponding to medium GT (70–74 °C), and grades 6 and 7 corresponding to low GT (< 70 °C) (Saif et al. 2003).

Cooking quality is mainly regulated by heredity, and is easily affected by environmental factors. To breed varieties with desirable cooking quality, lots of effort have been put into the genetic study of cooking quality. Some important and high-effect genes have been successfully cloned and applied in molecular breeding. The *Wx* gene located on chromosome 6 encodes the granule-bound starch synthase I (GBSSI) to catalyse amylose synthesis and mainly controls AC. Different alleles of *Wx*, including *wx*, *Wx*^*a*^, *Wx*^*b*^, *Wx*^*in*^, *Wx*^*op*^, *Wx*^*mq*^, *Wx*^*mp*^, *Wx*^*hp*^ and so on, often determine the level of AC (Wang et al. 1995; Sato et al. 2002; Mikami et al. 2008; Liu et al. 2009; Tian et al. 2009; Yang et al. 2013; Zhang et al. 2019b). In addition to controlling AC, the *Wx* gene also determines GC. The main effect QTL of GC has been confirmed to be located at the *Wx* site (Su et al. 2011). Besides, the *Wx* gene has also been shown to have a minor effect on GT (Tian et al. 2009). The *ALK* gene, which encodes the soluble starch synthase IIa (SSII-3) on chromosome 6, is considered to be the major effect gene for GT (Tan et al. 1999; Septiningsih et al. 2003), and also has an influence on GC (Gao et al. 2011). Some transcriptional regulatory factors affect cooking quality by regulating the expression of starch synthase genes. For example, *OsbZIP58* specifically binds to the ACGT motif in the promoter of *Wx* gene and enhances its expression. The null mutant of *OsbZIP58* is chalky and its total starch and AC are decreased (Wang et al. 2013). *OsMADS7* is a heat-induced gene that regulates AC synthesis by enhancing *Wx* gene expression (Zhang et al. 2018).

In addition to the major genes and transcriptional regulatory genes that have been cloned, quantitative trait loci (QTLs) of cooking quality are also important aspects to study their regulatory mechanisms. Some populations derived from individual parental crosses have been used to identify QTLs for cooking quality. For example, the *qSAC3* is a newly identified QTL from *indica* rice 93 − 11. And the introgression of *qSAC3* leads to an increased AC in *Japonica* rice Nipponbare and Nangeng 9108 under all tested growth environments (Zhang et al. 2019c). However, most QTLs could not be repeatedly identified in different environments, except for the major genes *Wx* and *ALK*, which have been consistently detected from various segregating populations of different cross-combinations across conditions (Wang et al. 2007; Liu et al. 2011; Hsu et al. 2014).

Recently, genome-wide association study (GWAS) has been widely used to identify QTLs for complex traits, such as rice quality (Qiu et al. 2021; Huo et al. 2023). Using 419 rice landraces core germplasm collections, one QTL for GC and one QTL for GT were detected by GWAS, which were co-located with *Wx* and *ALK*, respectively (Yang et al. 2018). Three QTLs were identified related to the elongation of cooked grain by bulk-segregant analysis and whole-genome sequencing based on an F_2_ population segregated for grain elongation as well as AC and GT (Arikit et al. 2019). Among them, *qGE4.1* is located near starch branching enzyme IIa (SBEIIa), and *qGE6.1* and *qGE6.2* are located near starch synthase IIa (SSIIa) and starch branching enzyme III (SBEIII), respectively. Based on 760 accessions from the 3 K Rice Genomes Project, 14 QTLs associated with cooked rice elongation were identified by GWAS, among which *qRED6.1* is co-located with *Wx.* Three genes (*LOC_Os06g43670*, *LOC_Os06g43680* and *LOC_Os06g43710*) are identified by haplotype analysis as possible candidate genes for *qREI6.4* (Qiu et al. 2021). Although progress has been made in the identification of QTLs related to cooking quality in rice, most QTLs are difficult to detect in different environments and are difficult to apply to molecular breeding. Therefore, it is necessary to further explore stable QTLs related to cooking quality and explore the genes of them.

In this study, the AC, GC and ASV of 450 diverse accessions were evaluated. A total of 54 QTLs were identified by GWAS including 25 QTLs for AC, 12 QTLs for GC and 17 QTLs for ASV. The *Wx* gene for AC and GC, and the *ALK* gene for ASV were identified in every population across two environments. 10 QTLs could be identified by the same population in both environments. Six QTLs were co-localized with the reported QTLs or cloned genes, and 48 QTLs were newly identified in the present study. Based on the linkage disequilibrium, RNA-sequencing and haplotype analysis, the E3 ubiquitin ligase gene *OsRING315* was considered as the candidate gene for both *qAC9-2* and *qGC9-2*. The expression level of *OsRING315* was related to AC and GC. *OsRING315* was differentiated between *indica* and *japonica*, and accessions with different haplotypes of *OsRING315* showed various AC and GC. This study reveals new insights for the genetic basis of cooking quality and provides a promising target for high-quality rice breeding.

## Materials and Methods

### Plant Materials

The 450 rice accessions used for phenotype evaluation, subpopulation comparison and GWAS were selected from RDP2 (McCouch et al. 2016) including 300 *indica* and 150 *japonica* rice. Details of the 450 accessions were listed in Table S1.

### Phenotypic Evaluation of AC, GC and ASV

The 450 rice accessions were planted in the experimental fields of Guangzhou (2016GZ) and Yangjiang (2018YJ) in Guangdong Province, China, in the second cropping season in 2016 and 2018, respectively. The seeds were sown at the end of July and transplanted in the mid-August. Since the growth period of each accession was different, the seeds were harvested 35 days after flowering. The seed harvesting began at the end of October and all seeds were harvested by the end of November. The rice planting, seed harvesting and storage referred to our previous reports (Huo et al. 2023). For the evaluation of AC, GC and ASV, 20 g grains were de-husked by a huller (JLG-III, Chengdu, China) and milled by a polisher (JNM, Chengdu, China). Then the milled grains were grinded by a grinder (LM3100, Stockholm, Sweden). The evaluation of AC was preprocessed according to the standard (GB/T 15,683 − 2008, China) and then determined by an automatic amylose analyzer (Futura-II, Frepillon, France). GC and ASV were measured according to the standard (GB/T 22,294 − 2008, China) and (NY/T 83-1988, China), respectively. All measurements were conducted with three independent samples, and the average values were used for subsequent analysis.

### GWAS and QTL Delimitation

GWAS was conducted as described in our previous study (Yang et al. 2021; Wang et al. 2023) but with slight modifications. To maximize the inclusion of samples while ensuring complete phenotypic data, we executed a tailored approach by conducting SNP filtering and GWAS separately for each phenotype within two distinct subpopulations (*indica* and *japonica*) as well as the whole population. Samples possessing complete phenotypic data in both environments were selectively retained. Briefly, SNPs were filtered using the criteria of having less than 10% of missing data and minor allele frequency (MAF) > 0.05. In the *indica* population, the sample sizes for AC, ASV, and GC were 290, 283, and 275, respectively, with corresponding SNP numbers being 1,516,071, 1,513,020, and 1,512,883. Meanwhile, the *japonica* population exhibited smaller sample sizes of 143, 134, and 144 for AC, ASV, and GC, respectively, with the SNP numbers recorded as 1,203,219, 1,171,922, and 1,196,152. Furthermore, when considering the data aggregated across both subpopulations, labeled as ‘whole’, the sample numbers for AC, ASV, and GC increased to 432, 416, and 418, respectively. This aggregation results in the highest observed SNP numbers across all categories, with counts of 1,686,487 for AC, 1,689,029 for ASV, and 1,691,932 for GC. The multiple loci mixed model (MLMM) was used for GWAS, and the principal component was set to 3 in GAPIT (Wang and Zhang 2021). Manhattan and QQ plots were produced by R-package CMplot (Yin et al. 2021). The linkage disequilibrium (LD) and LD blocks were calculated and visualized by LDBlockShow (Dong et al. 2021). The “LD decay distance” is defined as the physical span where linkage disequilibrium reduces to half its maximum value. In this study, a 200 kb distance, computed via PopLDdecay (Zhang et al. 2019a), was applied as the benchmark for the flanking region length around the target QTL. The GEC software was utilized to determine significant *p*-value thresholds, with a recommended cut-off threshold of 0.00001 for *p*-values as suggested (Li et al. 2012). A locus qualified as a potential associated QTL if flanked by two or more significant SNPs (*p* < 0.00001) within a 200 kb radius of a significant SNP. Potential functional genes were identified within the same LD block of significant QTL.

### RNA Sequencing

To identify candidate genes for *qGC9-2* and *qAC9-2*, RNA sequencing was employed to investigate the gene expression differences among individuals with significant variations in GC and AC, who also have different allelic types. Two groups, each containing 10 accessions, were distinguished: one group possessesed the C allele associated with lower GC, and the other carried the T allele linked to higher GC at the position Chr09: 14,724,296 for *qGC9-2*. Furthermore, two groups of 20 accessions were chosen for *qAC9-2*, where one exhibited the G allele correlated with higher AC and the other exhibited the T allele associated with lower AC at Chr09: 14,776,728. Two independent spikes without dehulling, serving as two replicates, were sampled from every accession on the 15th day after flowering. The total RNA was extracted individually extracted from each tissue and sequenced by the Berry Genomics (Beijing, China). The libraries with 150-bp paired-end (PE) reads were prepared for sequencing on an Illumina HiSeq X Ten platform. The short reads were processed using fastp (Chen et al. 2018). The Nipponbare genome (MSU v7.0) was used as a reference for reads mapping (Kawahara et al. 2013). Only uniquely mapped paired-end reads were retained for read counting of the genes by featureCounts (Liao et al. 2014). Differential gene expression analysis was performed with DEseq2 (Love et al. 2014). The differentially expressed genes were identified according to the criteria of adjusted *p* value < 0.05 and a fold change (FC) cut-off of 0.67 and 1.5.

### Real-Time PCR Analysis

The samples for RNA sequencing were used to confirm the expression patterns of *OsRING315* in this study. The cDNA synthesis, qRT-PCR and internal reference gene were conducted according to the published study (Yang et al. 2023). Briefly, the cDNA was synthesized using the PrimeScriptTM RT reagent kit (Takara, Japan). The qRT-PCR analysis was performed by qRT-PCR (Biorad CFX96, Pleasanton, CA, USA). All reactions were repeated three times. The primer sequences for qRT-PCR of *OsRING315* were 5’-GTATGCTCATCGGGCTTGTG-3’ and 5’-AGGCCTAGTAGGTGCAGTGTA-3’. The *EF1α* gene was used as the reference gene.

### Data Analysis and Image Production

Statistical comparison was conducted by the *t*-test. Principal component analysis (PCA) and Pearson correlation analysis were both conducted using base functions in *R* language. The *R* package geneHapR was utilized to compute and illustrate the frequency distribution of haplotypes across different regions of the world (Zhang et al. 2023).

## Results

### Phenotypic Variations of AC, GC and ASV in 450 Rice Accessions

**Fig. 1:**
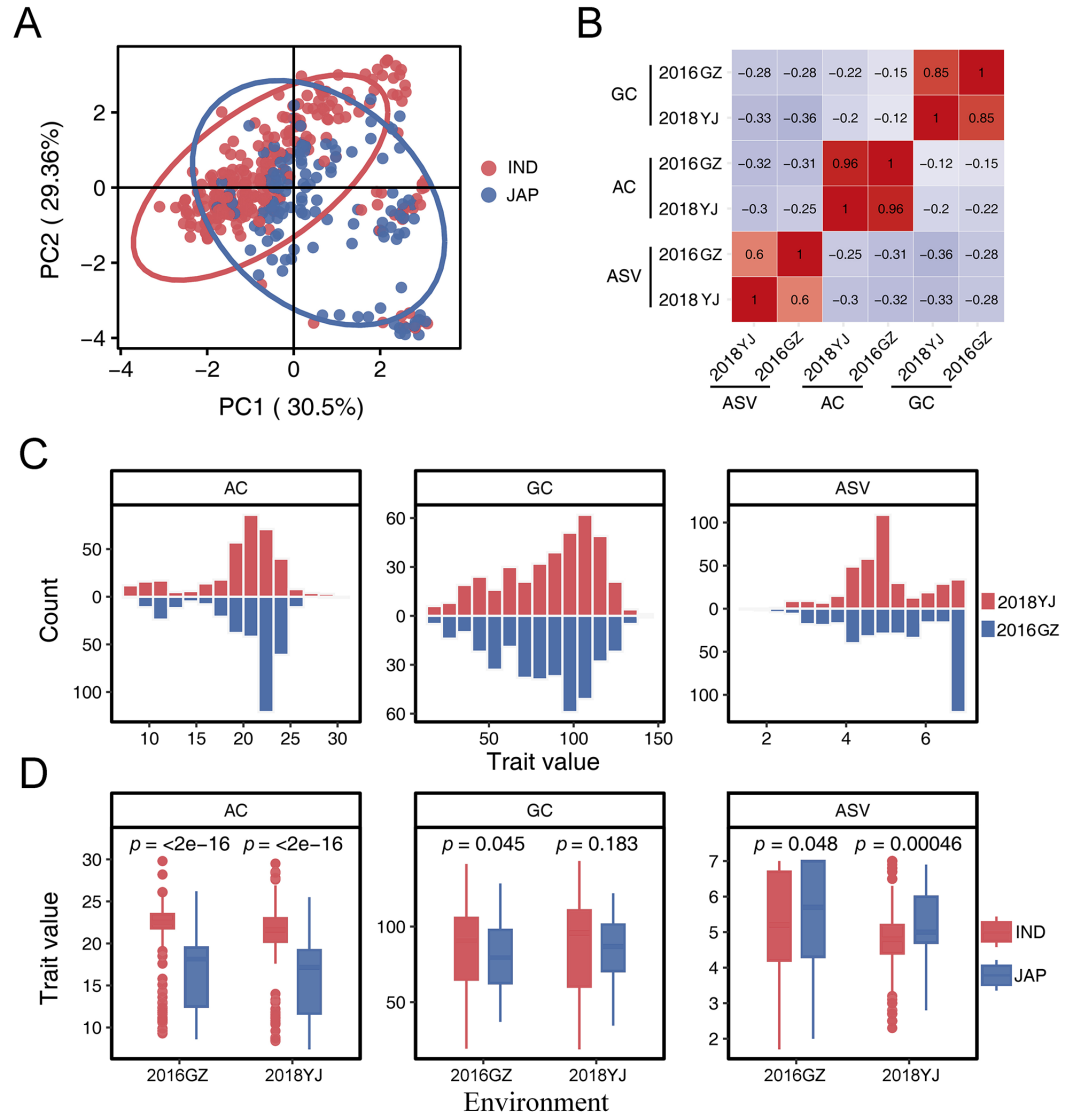
The phenotypic distribution and comparison of 450 rice accessions used in this study. (**A**) PCA (principal component analysis) for the 450 rice accessions. (**B**) Correlations between the three gain quality traits measured in 2016GZ and 2018YJ. Pearson correlation coefficients range from − 1 to 1, corresponding to a gradient from blue to red. (**C**) Phenotypic distribution of the three traits in the two environments. (**D**) Phenotypic comparison between *indica* and *japonica* accessions. Statistical comparison was conducted by the two-tailed *t*-test. The 2016GZ and 2018YJ represent two environments. AC: Amylose content, GC: gel consistency, ASV: alkali spreading value, IND: *indica*, JAP: *japonica*

PCA analysis showed that the 300 *indica* accessions and 150 *japonica* accessions could be roughly separated with some *indica* and *japonica* accessions clustered together (Fig. 1A), which is consistent with the finding based on evolutionary analysis in our previous study (Huo et al. 2023). The three traits (AC, GC and ASV) of the 450 rice accessions were evaluated in two environments (2016GZ and 2018YJ). The phenotype correlations of every trait between two environments were high with correlation coefficients of 0.96, 0.85 and 0.60 for AC, GC and ASV, respectively (Fig. 1B). There were negative correlations between the three traits. The negative correlation between GC and ASV is the highest with the correlation coefficient of -0.33 in 2018YJ (Fig. 1B). The phenotypic distribution of the three traits showed large variations in the two environments (Fig. 1C and Table S2). Relative to *japonica* rice, *indica* rice exhibited greater variability across the three phenotypes in both environments, potentially attributable to the more accessions of *indica* subpopulation in this study (Fig. 1D and Table S2). The *indica* rice showed higher AC and lower ASV than that of *japonica* rice. The difference of GC between the two subpopulations was observed in 2016GZ, but not in 2018YJ (Fig. 1D).

### QTLs Mapping by GWAS

**Fig. 2:**
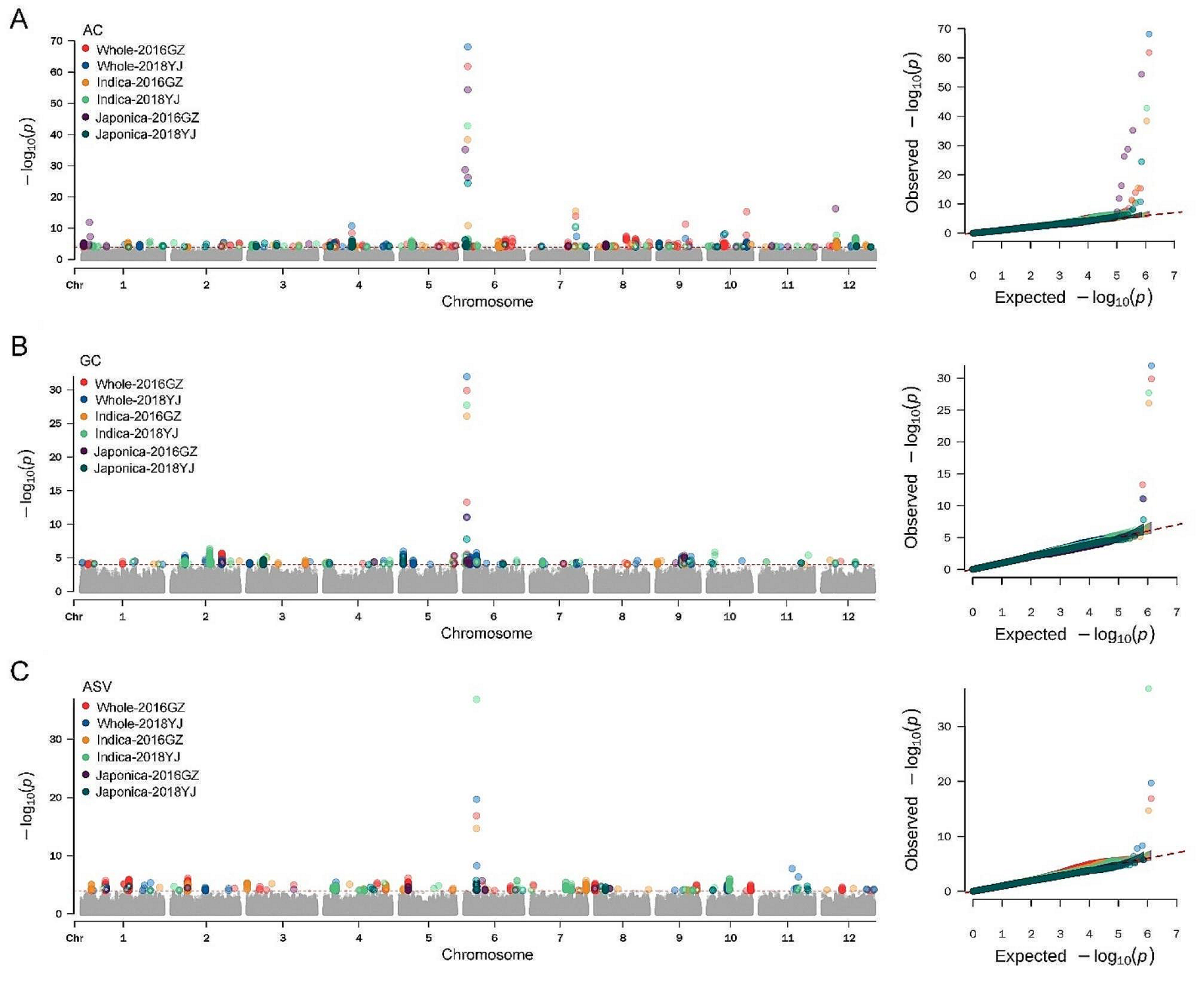
Genome-wide association study for AC, GC and ASV. (**A**), (**B**) and (**C**) represent the GWAS result for AC, GC and ASV, respectively

A total of 85 loci (41 loci for AC, 20 loci for GC and 24 loci for ASV) were identified in both environments. Among them, 41 loci (21 loci for AC, 10 loci for GC and 10 loci for ASV) were identified by the whole population, 26 loci (10 loci for AC, five loci for GC and 11 loci for ASV) were identified by the *indica* population, and 18 loci (10 loci for AC, five loci for GC and three loci for ASV) were identified by the *japonica* population. The co-localized loci identified by different populations or different environments for the same trait were defined as one QTL. Finally, a total of 54 QTLs were identified, including 25 QTLs for AC, 12 QTLs for GC and 17 QTLs for ASV (Fig. 2; Table 1).

Four QTLs for AC (*qAC4-2*, *qAC6-2, qAC7-2* and *qAC12-2*), four QTLs (*qGC5-1*, *qGC5-2*, *qGC6-1* and *qGC9-2*) for GC, and three QTLs (*qASV2-1*, *qASV6-1* and *qASV6-2*) for ASV can be identified in two or three populations, while other QTLs can only be identified in one population (Table 1). Within the same population, 10 QTLs were consistently identified in both environments, namely *qAC4-2*, *qAC6-1*, *qAC6-2*, *qAC7-2*, *qAC9-2*, *qAC10-1*, *qAC10-3* and *qAC12-2* for AC, *qGC6-1* for GC, and *qASV6-1* for ASV (Table 1).

**Table 1:** QTLs identified in the present study

QTL	Chromosome	Environment	Population	Position	*P* value	QTL/Gene	ref
**AC**							
*qAC1-1*	1	2016GZ	*Japonica*	1,028,922	4.42E-06		
*qAC1-2*	1	2016GZ	*Japonica*	3,993,329	1.34E-12		
*qAC1-3*	1	2016GZ	*Japonica*	4,404,404	4.78E-08		
*qAC1-4*	1	2016GZ	*Indica*	24,085,259	3.63E-06		
*qAC4-1*	4	2016GZ	Whole	5,779,943	2.68E-06		
*qAC4-2*	4	2018YJ	Whole	14,068,488	1.85E-11		
	4	2016GZ	Whole	14,136,203	3.53E-09		
	4	2018YJ	*Japonica*	14,178,161	4.92E-07		
*qAC5-1*	5	2018YJ	*Indica*	6,049,007	8.77E-07		
*qAC6-1*	6	2016GZ	*Japonica*	407,524	6.64E-36		
	6	2018YJ	*Japonica*	407,524	5.12E-07		
*qAC6-2*	6	2016GZ	*Indica*	1,644,116	4.18E-39	*Wx*	Wang et al. 1995
	6	2018YJ	*Indica*	1,644,116	1.59E-43		
	6	2016GZ	Whole	1,731,808	1.53E-62		
	6	2018YJ	Whole	1,731,808	7.29E-69		
	6	2016GZ	*Japonica*	1,731,808	4.15E-55		
	6	2018YJ	*Japonica*	1,731,808	3.80E-25		
*qAC6-3*	6	2016GZ	*Indica*	17,602,731	1.38E-06		
*qAC6-4*	6	2016GZ	Whole	21,445,577	3.12E-07		
*qAC6-5*	6	2016GZ	Whole	24,813,900	1.88E-07		
*qAC7-1*	7	2016GZ	Whole	19,026,812	3.18E-06		
*qAC7-2*	7	2018YJ	Whole	22,950,478	4.83E-11		
	7	2016GZ	Whole	23,062,536	1.31E-14		
	7	2016GZ	*Indica*	23,068,774	3.55E-16		
	7	2018YJ	*Indica*	23,068,774	2.44E-11		
*qAC7-3*	7	2018YJ	Whole	23,498,976	4.27E-08		
*qAC8-1*	8	2016GZ	Whole	16,052,546	3.25E-08		
*qAC8-2*	8	2016GZ	Whole	20,879,076	2.56E-07		
*qAC9-1*	9	2018YJ	Whole	1,082,767	5.14E-06		
*qAC9-2*	9	2018YJ	Whole	14,774,740	2.17E-07		
	9	2016GZ	Whole	14,776,728	5.03E-12		
*qAC10-1*	10	2016GZ	Whole	7,972,845	9.65E-06		
	10	2018YJ	Whole	7,972,845	1.39E-08		
*qAC10-2*	10	2018YJ	*Japonica*	8,595,337	6.01E-09		
*qAC10-3*	10	2016GZ	Whole	20,045,839	5.07E-16		
	10	2018YJ	Whole	20,045,839	2.01E-06		
*qAC12-1*	12	2016GZ	*Japonica*	6,761,997	5.49E-17		
*qAC12-2*	12	2016GZ	*Indica*	7,180,076	1.09E-06		
	12	2018YJ	*Indica*	7,180,076	1.61E-08		
	12	2018YJ	Whole	7,369,409	2.85E-06		
*qAC12-3*	12	2018YJ	*Indica*	17,068,567	1.03E-07		
**GC**							
*qGC2-1*	2	2018YJ	Whole	7,340,632	4.25E-06		
*qGC2-2*	2	2018YJ	*Indica*	20,252,201	4.14E-07		
*qGC2-3*	2	2016GZ	Whole	26,574,491	2.00E-06		
*qGC5-1*	5	2018YJ	Whole	2,246,348	1.01E-06		
	5	2018YJ	*Japonica*	2,249,302	6.24E-06		
*qGC5-2*	5	2016GZ	Whole	28,815,688	4.64E-06	*OsAGPL3*	Ohdan et al. (2005)
	5	2016GZ	*Japonica*	28,815,688	5.25E-06		
*qGC6-1*	6	2016GZ	*Japonica*	1,731,808	9.47E-12	*Wx*	Wang et al. 1995
	6	2018YJ	*Japonica*	1,731,808	1.66E-08		
	6	2016GZ	Whole	1,768,998	1.30E-30		
	6	2018YJ	Whole	1,768,998	1.12E-32		
	6	2016GZ	*Indica*	1,768,998	8.36E-27		
	6	2018YJ	*Indica*	1,768,998	1.97E-28		
*qGC6-2*	6	2018YJ	Whole	3,631,741	6.51E-06		
*qGC6-3*	6	2018YJ	Whole	6,832,717	1.68E-06	*qGE6.1*	Arikit et al. 2019
*qGC9-1*	9	2018YJ	Whole	13,391,560	5.86E-06	*qREI6.2*	Qiu et al. 2021
*qGC9-2*	9	2016GZ	*Japonica*	14,656,818	4.31E-06		
	9	2016GZ	*Indica*	14,724,296	7.37E-06		
*qGC9-3*	9	2018YJ	Whole	18,150,109	6.09E-06		
*qGC10-1*	10	2018YJ	*Indica*	3,959,669	1.41E-06		
**ASV**							
*qASV1-1*	1	2016GZ	*Indica*	5,454,172	8.24E-06		
*qASV1-2*	1	2016GZ	Whole	12,772,887	1.78E-06		
*qASV1-3*	1	2016GZ	Whole	24,989,416	1.11E-06		
*qASV2-1*	2	2016GZ	Whole	8,730,258	6.77E-07		
	2	2016GZ	*Indica*	8,730,258	4.77E-06		
*qASV3-1*	3	2016GZ	*Indica*	61,819	4.88E-06		
*qASV4-1*	4	2016GZ	Whole	27,707,649	4.46E-06		
*qASV4-2*	4	2018YJ	*Indica*	31,543,034	5.19E-06		
*qASV4-3*	4	2016GZ	*Indica*	32,168,337	8.59E-06		
*qASV5-1*	5	2016GZ	Whole	4,665,090	6.61E-07		
*qASV6-1*	6	2018YJ	*Japonica*	6,581,196	1.80E-06	*ALK; SSIIa*	Septiningsih et al. 2003
	6	2016GZ	Whole	6,710,536	1.35E-17		
	6	2016GZ	*Indica*	6,710,536	2.07E-15		
	6	2018YJ	Whole	6,752,887	1.97E-20		
	6	2018YJ	*Indica*	6,752,887	1.35E-37		
	6	2016GZ	*Japonica*	6,752,887	5.20E-06		
*qASV6-2*	6	2016GZ	Whole	9,666,234	7.27E-06		
	6	2016GZ	*Japonica*	9,666,234	1.94E-06		
*qASV6-3*	6	2018YJ	*Indica*	27,839,664	1.73E-06		
*qASV7-1*	7	2018YJ	*Indica*	18,343,061	3.31E-06		
*qASV7-2*	7	2016GZ	*Indica*	28,884,999	1.76E-06		
*qASV8-1*	8	2016GZ	Whole	247,342	5.10E-06		
*qASV10-1*	10	2018YJ	*Indica*	11,548,336	8.47E-07		
*qASV11-1*	11	2018YJ	Whole	17,250,181	1.63E-08		

Compared with the previous studies, six QTLs identified in this study were co-localized with the reported QTLs or cloned genes (Table 1). In particularly, *qAC6-2* and *qGC6-1* were co-located with *Wx*, a major gene controlling AC and GC (Wang et al. 1995); *qASV6-1* was co-located with *ALK*, a major gene controlling ASV (Septiningsih et al. 2003); *qGC5-2* was co-located with *OsAGPL3*, an ADP-glucose pyrophosphorylase gene controlling starch content (Akihiro et al. 2005). *qGC6-3* and *qGC9-1* were co-located with *qGE6.1* (Arikit et al. 2019) and *qREI6.2* (Qiu et al. 2021), respectively. The other 48 QTLs were newly identified in the present study (Table 1).

### Region and Phenotype Analysis of *qAC9-2* and *qGC9-2*

**Fig. 3:**
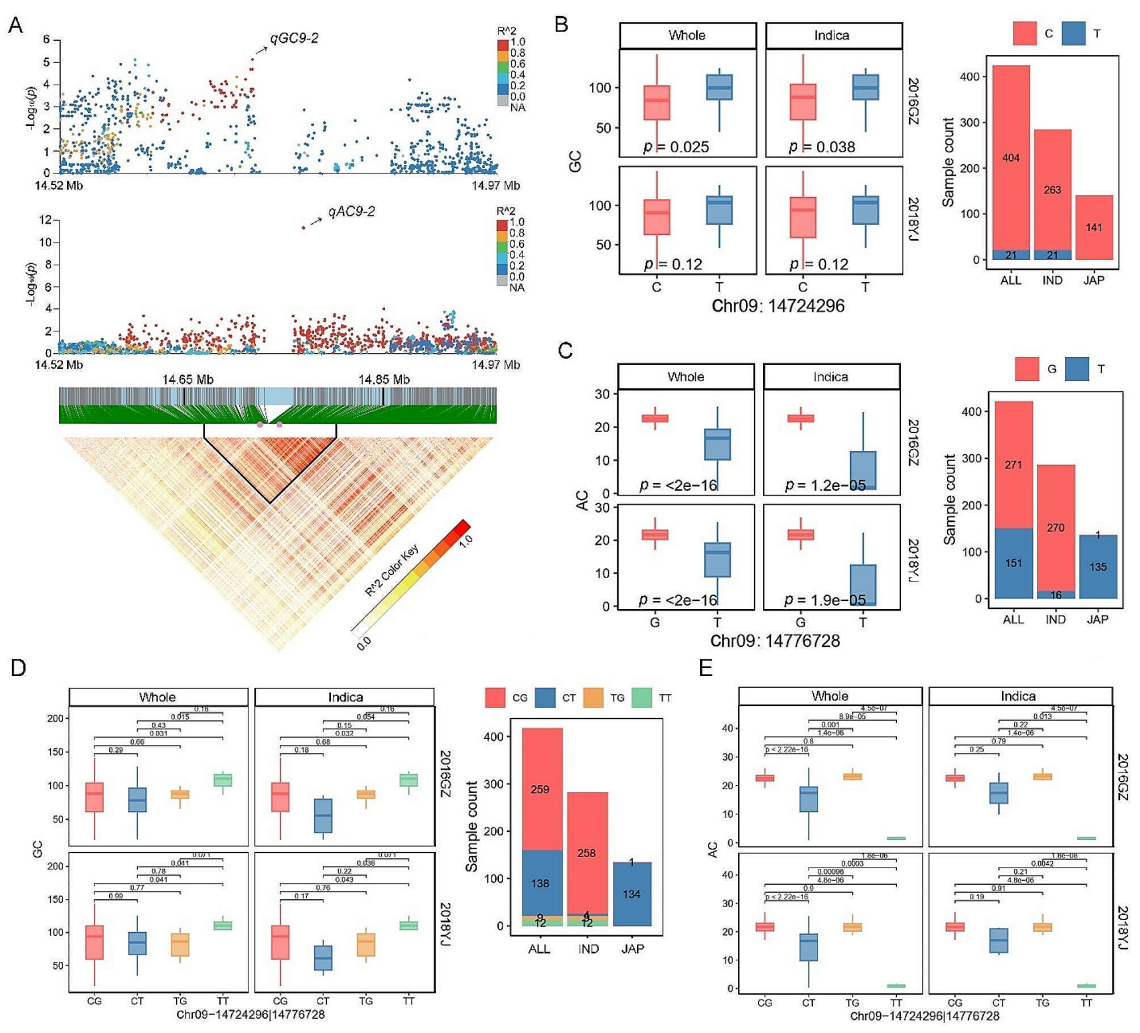
Genomic region and phenotypic comparisons for *qGC9-2* and *qAC9-2*. (**A**) The local Manhattan plot and the linkage disequilibrium heatmap for *qGC9-2* and *qAC9-2*. (**B**) Phenotypic comparisons and haplotype distributions for *qGC9-2*. (**C**) Phenotypic comparisons and haplotype distributions for *qAC9-2*. (**D**) Phenotypic comparisons of GC and haplotype distributions for combinations of the two significant SNP corresponding to *qAC9-2* and *qGC9-2*. (**E**) Phenotypic comparisons of AC for combinations of the two significant SNP corresponding to *qAC9-2* and *qGC9-2*. Statistical comparison was conducted by the two-tailed *t*-test

For the newly identified QTLs in the present study, the *qAC9-2* for AC was identified in the whole population in both environments and showed a relatively significant *P*-value (Table 1). Additionally, the *qGC9-2* for GC was identified in the *japonica* and *indica* population in 2016GZ, respectively (Table 1). The linkage disequilibrium (LD) analysis indicated that *qAC9-2* and *qGC9-2* were co-located (Fig. 3A). The most significant SNPs, specifically Chr09: 14,776,728 for *qAC9-2* and Chr09: 14,724,296 for *qGC9-2*, were separated by a mere 50 kb. A linkage block of approximately 200 kb (from 14.65 to 14.85 Mb) was identified as the putative region for *qAC9-2* and *qGC9-2* (Fig. 3A). We then explored candidate genes for the two tightly linked QTLs.

We discovered that the most significant SNP for *qGC9-2* possessed a biased segregation frequency of the minor allele (T), whereas the most significant SNP for *qAC9-2* displayed characteristics of *indica-japonica* differentiation in two alleles (G and T) (Fig. 3B and C). For *qGC9-2* loci, the GC was different between accessions with diverse alleles in the whole and *indica* population in 2016GZ, and no such difference was observed in 2018YJ (Fig. 3B). While there was no variation in the position of *qGC9-2* locus in the *japonica* accessions. For *qAC9-2* loci, the AC were different between accessions with diverse alleles in the whole and *indica* population across the two environments. Similarly, the variations in the position of *qAC9-2* locus were almost all the T haplotype (Fig. 3C).

In order to investigate the linkage relationship between the two QTLs, four haplotypes (designated as CG, CT, TG, and TT) were derived by combining the different alleles of the two SNPs (Fig. 3D). The accessions with TT haplotype showed higher GC than that of accessions with CG haplotype in the whole and *indica* population in the two environments. The accessions with TT haplotype also showed higher GC than that of accessions with CT haplotype in the whole population in 2016GZ, and also in the whole and *indica* population in 2018YJ (Fig. 3D). Interestingly, the accessions with TT haplotype showed the lowest AC than that of accessions with the other three haplotypes. The average AC of these accessions with TT haplotype were only 3.35% in 2016GZ and 3.80% in 2018YJ (Table S3). The accessions with CT haplotype showed lower AC than that of accessions with CG or TG in the whole population in the two environments (Fig. 3E).

### Candidate gene Analysis of *qAC9-2* and *qGC9-2*

**Fig. 4:**
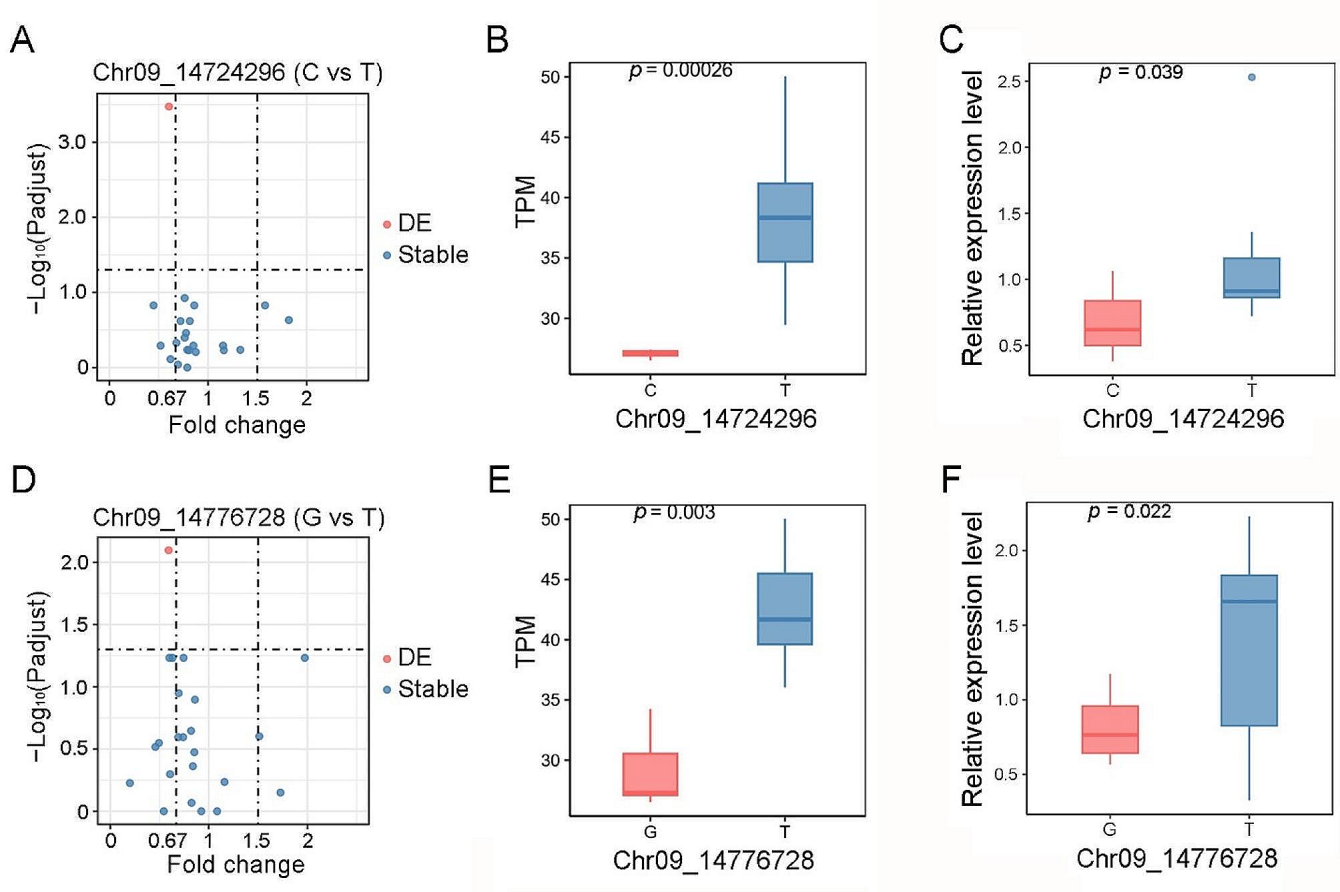
Expression analysis of candidate genes for *qGC9-2* and *qAC9-2*. (**A**) In the LD block of *qGC9-2*, genes with significant differential expression have been identified between samples corresponding to each of the two alleles of SNP (Chr09_14724296). Red dot has been defined as significantly differentially expressed gene (DE), while blue dots represent stably expressed genes. (**B**) The expression of *LOC_Os09g24650* (DE gene in panel A) in accessions with contrasting haplotypes of *qGC9-2*. (**C**) The relative expression level of *LOC_Os09g24650* in accessions with contrasting SNP (Chr09_14724296) by qRT-PCR. (**D**) In the LD block of *qAC9-2*, genes with significant differential expression have been identified between samples corresponding to each of the two alleles of SNP (Chr09_14776728). Red dot has been defined as significantly differentially expressed gene (DE), while blue dots represent stably expressed genes. (**E**) The expression of *LOC_Os09g24650* (DE gene in panel D) in accessions with contrasting haplotypes of *qAC9-2*. (**F**) The relative expression level of *LOC_Os09g24650* in accessions with contrasting SNP (Chr09_14776728) by qRT-PCR. Statistical comparison was conducted by the two-tailed *t*-test

There were 22 annotated genes within the *qAC9-2* and *qGC9-2* region (Table S4) based on release 7 of the MSU Rice Genome Annotation Project (Kawahara et al. 2013). Since the grain filling stage is the key period for grain quality, and the differentially expressed genes during this stage may result in variant quality (Yang et al. 2022; Ma et al. 2023). To further localize the candidate genes, the spikes (15-day after flowering) of 10 accessions with lower GC (Chr09: 14,724,296, C allele) and 10 accessions with higher GC (Chr09: 14,724,296, T allele) were sampled for RNA sequencing, respectively. Only one gene (*LOC_Os09g24650*) was significant differentially expressed between the two sets of contrasting accessions (Fig. 4A). The expression level of *LOC_Os09g24650* in the accessions with lower GC were lower than that in accessions with higher GC (Fig. 4B). qRT-PCR assays confirmed that the expression patterns of *LOC_Os09g24650* were consistent with the RNA-sequencing result (Fig. 4C).Interestingly, only the same gene (*LOC_Os09g24650*) was differentially expressed between the two sets of contrasting accessions for *qAC9-2* (Fig. 4D). The *LOC_Os09g24650* exhibited significantly elevated expression levels in accessions with lower AC compared to those with higher AC (Fig. 4E). qRT-PCR assays also confirmed that the expression patterns of *LOC_Os09g24650* were consistent with the RNA-sequencing result (Fig. 4F). According to the genome annotation, *LOC_Os09g24650* encoding an E3 ubiquitin ligase belongs to the C3HC4-type RING zinc finger protein family. A previous study of global analysis for E3 ubiquitin ligases named *LOC_Os09g24650* as *OsRING315* (Wang et al. 2022).

### Haplotype and Distribution Analysis of *OsRING315*

**Fig. 5:**
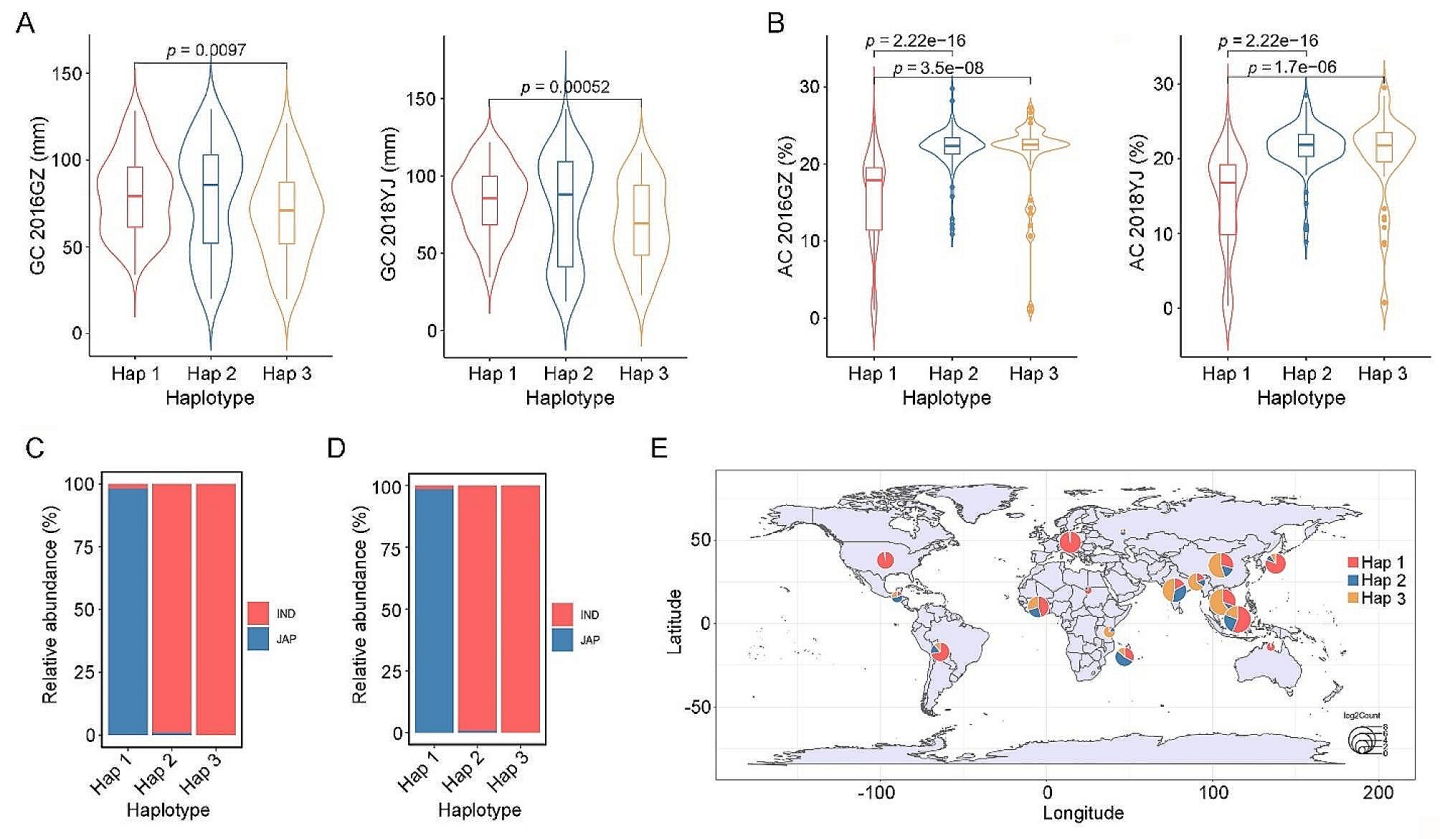
Haplotype and distribution analysis of *OsRING315*. (**A**) Comparison of GC among accessions harboring various haplotypes of *OsRING315*. (**B**) Comparison of AC among accessions harboring various haplotypes of *OsRING315*. Statistical comparison was conducted by the two-tailed *t*-test. (**C**) and (**D**) The distributions of haplotypes in our core sequenced germplasms and in the 3 K rice genomes, respectively. (**E**) The geographic distributions of the three haplotypes among 3 K rice genomes

To investigate the different haplotypes of *OsRING315*, we analyzed variations in the promoter (2000 bp from ATG), coding sequence (CDS) and 3’-untranslated region (3’ UTR) in the 450 rice accessions, which have been sequenced in our previous study (Wang et al. 2023). 26 SNPs in the promoter region, three SNPs in the CDS, and one SNP in 3’UTR were revealed. Three haplotypes were identified (Hap 1, Hap 2 and Hap 3) (Figure S1). We further associated the three haplotypes with GC and AC. The accessions with Hap 1 showed higher GC than that of accessions with Hap 3 in the two environments. No significant differences were observed in other pairwise comparisons among haplotypes (Fig. 5A). Notably, the accessions with Hap 1 showed lower AC than that of accessions with Hap 2 or Hap 3 in the two environments, while no difference in AC between Hap 2 and Hap 3 was detected (Fig. 5B).

We further analyzed the distribution of three haplotypes in the 450 rice accessions. Interestingly, almost all *japonica* accessions (98.1%) but few *indica* accessions (1.9%) harbored Hap 1, and almost all *indica* accessions harbored Hap 2 or Hap 3 (Fig. 5C). This result was consistent with the observation in the 3 K rice genomes (Fig. 5D). In addition, an unbalanced geographical distribution of the three haplotypes in several rice regions was exhibited (Fig. 5E). The Hap1 was distributed in all major regions of the world. The accessions from Europe and North America mostly contained Hap 1. The Hap 2 and Hap 3 were found primarily in Asia, Africa, and South America. However, the proportion of the Hap 2 and Hap 3 in different countries was various (Fig. 5E).

### Effect on AC and GC of Interactions between *OsRING315* and *Wx*

The *Wx* gene is the major gene determining AC and GC (Wang et al. 1995; Su et al. 2011). We further analyzed the effects on AC and GC of interactions between *OsRING315* and *Wx*. In the whole population, the AC and GC varied with the changed haplotypes of *Wx*, which further indicated that *Wx* is the main gene controlling AC and GC. Analysis of interactions between *OsRING315* and *Wx* showed that the AC was not affected by the various haplotypes of *OsRING315*, while the GC was affected by the various haplotypes of *OsRING315* (Figure S2). For example, in the *indica* population, when the haplotype of *Wx* was *Wx*^*a*^, the AC was not affected by the changed haplotype of *OsRING315*, while accessions with Hap 3 of *OsRING315* showed higher GC than that of accessions with Hap 2. When the haplotype of *Wx* was *Wx*^*iv*^, the AC was also not affected by the changed haplotype of *OsRING315*, while accessionss with Hap 3 of *OsRING315* showed lower GC than that of accessions with Hap 2.

## Discussion

Cooking quality is one of the key considerations for breeders and consumers. AC, GC and ASV are the three most commonly used indexes to evaluate cooking quality. Although the *Wx* is the major gene controlling AC and GC (Wang et al. 1995; Su et al. 2011), we found that the correlation coefficient between AC and GC is not substantial (Fig. 1B), indicating that more genetic factors regulate them. Besides the *Wx* gene, the *ALK* gene is considered to be the major gene for GT (Tan et al. 1999; Septiningsih et al. 2003), and also has an influence on GC (Gao et al. 2011). In this study, the *Wx* gene for AC and GC, and *ALK* gene for ASV were identified in every population across the two environments, indicating the accuracy of our GWAS. Meanwhile, the *Wx* gene and *ALK* gene had the most significant *P* value, which further indicated the main effect of the two genes on AC and GC, and ASV, respectively. At the same time, we identified some QTLs that can be repeatedly detected in different environments. However, more QTLs can only be detected in one environment (Table 1), suggesting that these QTLs were influenced by environments. These results indicate that the three traits are controlled by both major and minor genes and are susceptible to environmental influences.

Among the QTLs identified in this study, the *qAC9-2* for AC was identified in the whole population in both environments. The *qGC9-2* for GC, only 50 kb nearby *qAC9-2*, could be identified in the *japonica* and *indica* population in 2016GZ, respectively. The LD analysis defined the most significant SNPs of the two QTLs to the same interval (Fig. 3A). By integrating the two SNPs and phenotypes, we found that the SNPs differentiated between *indica* and *japonica* rice. The different SNP or combinations showed variable phenotypes (Fig. 3B ~ 3E). Rice varieties with good eating quality, such as *indica* rice in South China that has been released in recent years, tend to have low AC (12%~20%) and soft GC (> 60 mm) (Chen et al. 2023). At the *qAC9-2* and *qGC9-2* loci, we found that most of the accessions with TT haplotype were waxy rice (Table S3), and these accessions had a relatively high GC (Fig. 3D). Without considering the waxy rice, accessions with a relatively low AC (CT haplotype) did not have a relatively high GC, or even lower GC in the *indica* accessions (Fig. 3D). Therefore, the *qAC9-2* and *qGC9-2* identified in this study partly explain the weak correlation between AC and GC, which are mainly regulated by *Wx* gene. Interestingly, the T haplotype of *qGC9-2* existed only in *indica* rice (Fig. 3B). While the T haplotype of *qAC9-2* existed in both *indica* and *japonica* rice, but the proportion was lower in *indica* rice (5.59%) and higher in *japonica* rice (99.26%) (Fig. 3C). These results indicated that the *qAC9-2* and *qGC9-2* were differentiated between subpopulations, to some extent explaining the differences in GC and AC between *indica* and *japonica* rice.

To identify the genes underlying *qAC9-2* and *qGC9-2*, we delimited the two QTLs to a 200 kb region containing 22 genes based on the LD decay analysis (Fig. 3A and Table S4). Only one gene, *LOC_Os09g24650* (named as *OsRING315*), were differentially expressed between the accessions with of contrasting haplotype. *OsRING315* encodes the E3 ubiquitin ligase. Plant genomes encode approximately 1,500 E3 ubiquitin ligases, which are involved in regulating many biological processes in plants (Hua and Vierstra 2011). In rice, there are 1,515 E3 ubiquitin ligase genes, some of which are related to grain quality (Song et al. 2007; Wang et al. 2022). Therefore, *OsRING315* was considered as the candidate gene for both *qAC9-2* and *qGC9-2*. *OsRING315* was highly expressed in the accessions with T haplotype of *qAC9-2* and in the accessions with T haplotype of *qGC9-2* (Fig. 4). The T haplotype of *qAC9-2* and the T haplotype of *qGC9-2* corresponded to lower AC and higher GC, respectively (Fig. 3). Therefore, relative higher expression of *OsRING315* may be conducive to reducing AC and increasing GC. Further studies are needed to confirm the functions of *OsRING315* in GC and AC through gain or loss-of function analysis.

Three haplotypes of *OsRING315* were identified in this study. The Hap 1 was mainly found in *japonica* accessions and had lower AC. The Hap 2 and Hap 3 were mainly found in *indica* accesssions, which had higher AC. Meanwhile, the GC of accessions harboring Hap 1 was higher than that of accessions harboring Hap 3 (Fig. 5). Therefore, *OsRING315* differentiates in *indica* and *japonica* rice. The varing haplotypes of *OsRING315* also regulates the difference of GC and AC between *indica* and *japonica* rice. In addition, a small number of *indica* accessions also contained Hap 1 (Fig. 5), indicating that the Hap 1 from *japonica* rice had infiltrated or retained in *indica* rice. Interestingly, the interactions between *OsRING315* and *Wx* showed that the AC was not affected by the various haplotypes of *OsRING315*, while the GC was affected by the various haplotypes of *OsRING315* (Figure S2A). As an E3 ubiquitin ligase gene, how *OsRING315* interacts with *Wx* to regulate GC is worthy of further study. In general, the haplotypes and distribution of *OsRING315* provides valuable information for understanding the genetic basis of AC and GC in rice.

## Conclusion

In this study, 54 QTLs were identified for cooking quality. Six QTLs were co-localized with the reported QTLs or cloned genes. The newly identified *qAC9-2* for AC and *qGC9-2* for GC were co-located. The E3 ubiquitin ligase gene *OsRING315* was considered as the candidate gene for both *qAC9-2* and *qGC9-2*. The expression level of *OsRING315* was correlated with AC and GC. The haplotypes of *OsRING315* exhibited differentiation between *indica* and *japonica* rice varieties. Accessions with different haplotypes of *OsRING315* showed various AC and GC. This study reveals new insights for the genetic basis of cooking quality, and *OsRING315* can be used as a potential target for high-quality rice breeding.

### Electronic Supplementary Material

Below is the link to the electronic supplementary material.

Supplementary Material 1

Supplementary Material 2

## Data Availability

No datasets were generated or analysed during the current study. The raw RNA-seq data are now available at the National Genomics Data Center under the BioProject number PRJCA024709.

## Abbreviations

AC: amylose content
ASV: alkali spreading value
CDS: coding sequence
GBSSI: granule-bound starch synthase I
GC: gel consistency
GT: gelatinization temperature
GWAS: genome-wide association study
LD: linkage disequilibrium
MLMM: multiple loci mixed model
PCA: Principal component analysis
QTLs: quantitative trait loci
SBEIIa: starch branching enzyme IIa
SSII-3: soluble starch synthase IIa
SSIIa: starch synthase IIa
UTR: untranslated region
