# Supplementary material for: GWAS and Transcriptomic Analysis Identify OsRING315 as a New Candidate Gene Controlling Amylose Content and Gel Consistency in Rice
Source: Rice (N Y). 2024 Jun 8;17:38. doi: 10.1186/s12284-024-00718-8 (PMC11161452; doi:10.1186/s12284-024-00718-8)

**Figure S1**. Three haplotypes of *OsRING315* identified in this study.


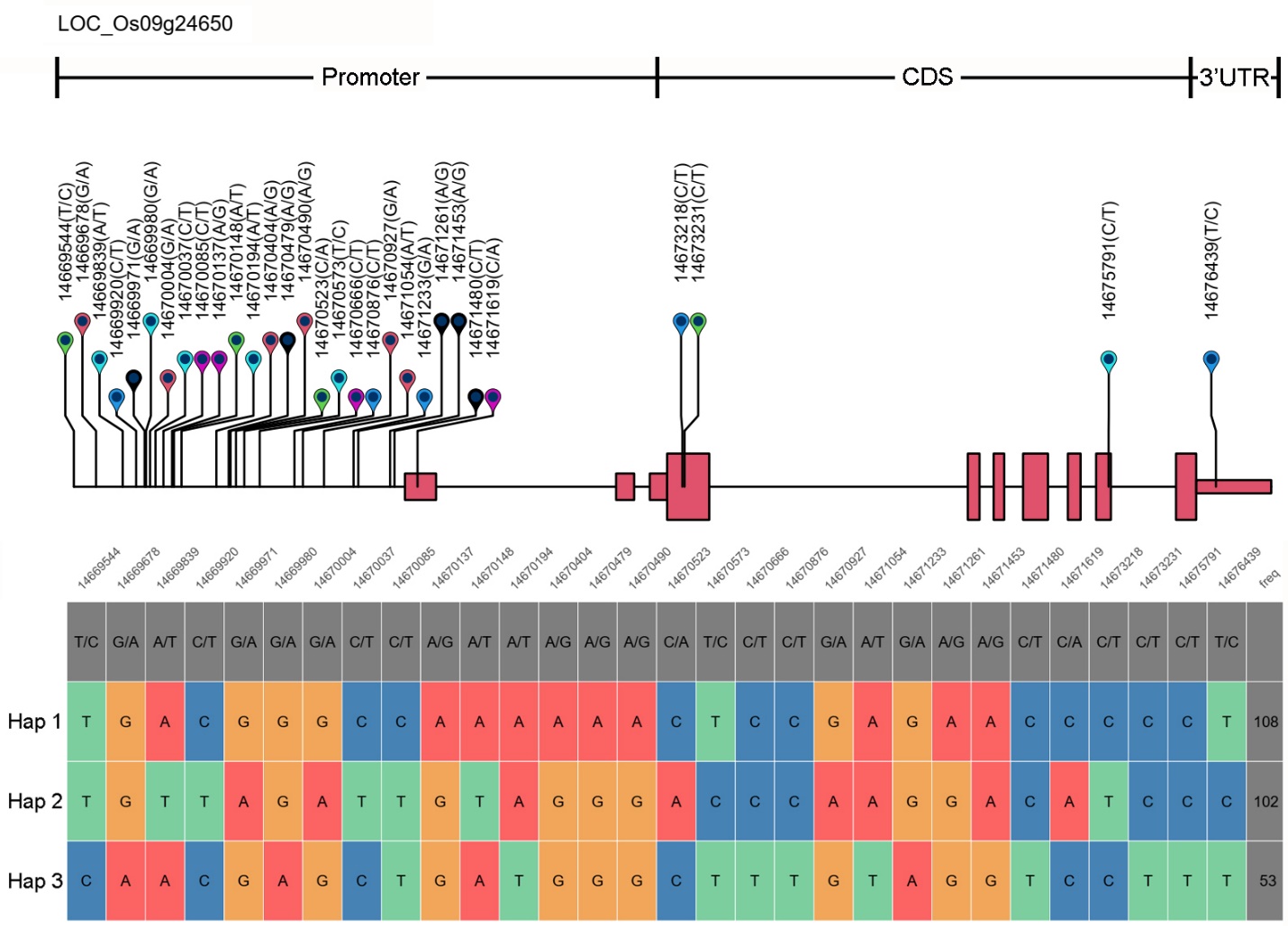


**Figure S2**. Effect on AC and GC of interactions between *OsRING315* and *Wx*. ALL: whole population, IND: *indica*, JAP: *japonica*. *wx*-Hap 1 represents accessions with both *wx* of *Wx* and Hap 1 of *OsRING315.*


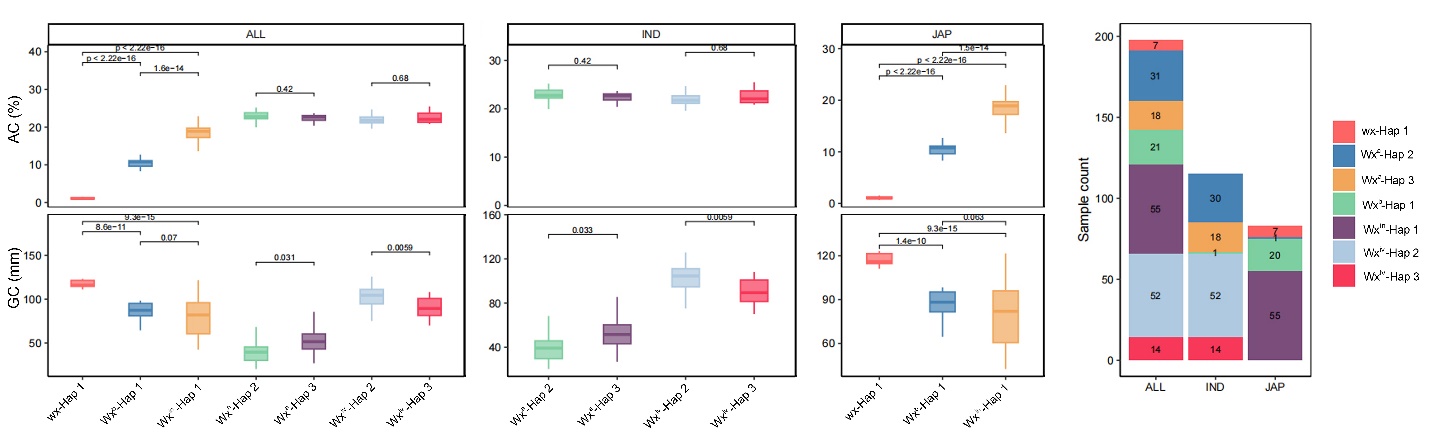

Supplement: Supplementary file 1 — Supplementary Material 1 [file 12284_2024_718_MOESM1_ESM.docx]
